# Functionality of Top-Rated Mobile Apps for Depression: Systematic Search and Evaluation

**DOI:** 10.2196/15321

**Published:** 2020-01-24

**Authors:** Chengcheng Qu, Corina Sas, Claudia Daudén Roquet, Gavin Doherty

**Affiliations:** 1 School of Computing and Communications Lancaster University Lancaster United Kingdom; 2 School of Computer Science and Statistics Trinity College Dublin Dublin Ireland

**Keywords:** mobile apps, depression, review, ethics, mHealth

## Abstract

**Background:**

In the last decade, there has been a proliferation of mobile apps claiming to support the needs of people living with depression. However, it is unclear what functionality is actually provided by apps for depression, or for whom they are intended.

**Objective:**

This paper aimed to explore the key features of top-rated apps for depression, including descriptive characteristics, functionality, and ethical concerns, to better inform the design of apps for depression.

**Methods:**

We reviewed top-rated iPhone OS (iOS) and Android mobile apps for depression retrieved from app marketplaces in spring 2019. We applied a systematic analysis to review the selected apps, for which data were gathered from the 2 marketplaces and through direct use of the apps. We report an in-depth analysis of app functionality, namely, screening, tracking, and provision of interventions. Of the initially identified 482 apps, 29 apps met the criteria for inclusion in this review. Apps were included if they remained accessible at the moment of evaluation, were offered in mental health–relevant categories, received a review score greater than 4.0 out of 5.0 by more than 100 reviewers, and had depression as a primary target.

**Results:**

The analysis revealed that a majority of apps specify the evidence base for their intervention (18/29, 62%), whereas a smaller proportion describes receiving clinical input into their design (12/29, 41%). All the selected apps are rated as suitable for children and adolescents on the marketplace, but 83% (24/29) do not provide a privacy policy consistent with their rating. The findings also show that most apps provide multiple functions. The most commonly implemented functions include provision of interventions (24/29, 83%) either as a digitalized therapeutic intervention or as support for mood expression; tracking (19/29, 66%) of moods, thoughts, or behaviors for supporting the intervention; and screening (9/29, 31%) to inform the decision to use the app and its intervention. Some apps include overtly negative content.

**Conclusions:**

Currently available top-ranked apps for depression on the major marketplaces provide diverse functionality to benefit users across a range of age groups; however, guidelines and frameworks are still needed to ensure users’ privacy and safety while using them. Suggestions include clearly defining the age of the target population and explicit disclosure of the sharing of users’ sensitive data with third parties. In addition, we found an opportunity for apps to better leverage digital affordances for mitigating harm, for personalizing interventions, and for tracking multimodal content. The study further demonstrated the need to consider potential risks while using depression apps, including the use of nonvalidated screening tools, tracking negative moods or thinking patterns, and exposing users to negative emotional expression content.

## Introduction

### Background

Depression is a major affective disorder with significant socioeconomic cost [1], affecting over 300 million people worldwide [2] across the life span [3]. However, access to treatment is problematic [4] given the acknowledged barriers such as high treatment cost, time constraints [4], geographical location [5], and stigma [4-7]. With over 90% worldwide penetration [8], mobile phones have significant potential to scale up the provision of interventions targeting depression [9]. They are especially useful to reach users who do not normally seek professional support, such as adolescents [10]. Prior work has already indicated a high user acceptance and effectiveness of mobile-delivered interventions for depression [11,12]. The number of mobile apps available on marketplaces offering treatment for depression has also been growing rapidly [9,13].

The apps available on mobile phone marketplaces provide access to a range of interventions targeting depression [14-16], which people can select and download to fit their needs [17]. Yet, users acting independently can only select apps based on information that is available at the point of download, ie, popularity, user ratings, or app descriptions provided on the marketplaces. Evidence for supporting assessment of the quality of an app, ie, structured description of its main features, evidence-based functionality, and potential risks, is not reflected in user ratings of apps [18,19]. Additionally, marketplaces do not require app developers to provide such information [20,21]. As a result, concerns have been raised regarding the lack of an evidence base for mental health apps [15,19,22] and poor regulation of the major mobile marketplaces [23-25] hosting them. Prior work [26] has also suggested the importance of having controlled clinical trials to determine the efficacy of new therapeutic treatments. In this newly established field of mobile health (mHealth) apps, most apps claim to be informed by evidence-based treatments rather than presenting rigorous evaluations of the app itself.

Besides efficacy, understanding patients (eg, their characteristics, needs, and behaviors) is also key for improving the uptake of apps [26,27]. Most human-computer interaction (HCI) studies on understanding [28-30] or supporting depression have focused on designing and evaluating mobile technologies in research contexts rather than marketplaces [31-33]. Scholarly work has also called for the evaluation of commercial apps for depression to support the effective development of the rapidly growing market of commercial apps [10,13,15]. However, such evaluations tend to focus in isolation on specific aspects such as ethics [34] and safety [35] or on specific interventions such as cognitive behavior therapy (CBT) or acceptance and commitment therapy (ACT) [10,19]. Moreover, previous evaluations tend to analyze app information from marketplaces without the actual experience of using the apps [15].

### Objectives

This paper addresses these limitations by focusing on a broader range of interventions and functionality of the top-rated apps for depression. Thus, we focused on the following research questions:

1. Which are the key functionalities of the top-rated apps for depression available on iPhone OS (iOS) and Android marketplaces?
2. Is this functionality described and delivered in a way that supports user privacy and safety?

## Methods

### Overview

This paper focuses on apps selected in spring 2019 from 2 major marketplaces, iOS and Android, whose analysis triangulates (1) reviewing app ratings on marketplaces to identify the top-rated apps for depression, (2) reviewing app descriptions on marketplaces, and (3) experimental evaluation through author interaction with the apps as expert HCI researchers [36,37].

### App Selection

**Figure 1:**
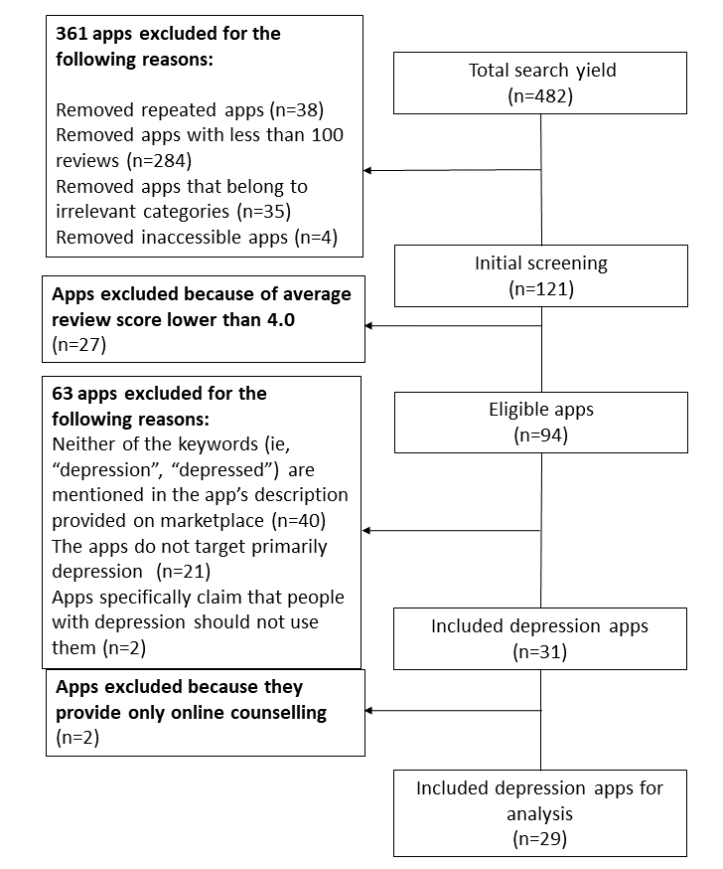
App extraction progress.

We now describe the selection process (Figure 1). The apps were initially identified through the 2 keywords “depression” and “depressed” entered into App Crawler and Google Play search engines. A script was used [38] to extract all the apps shown in the search results. The script automatically downloaded information for each app from its marketplace, including name, category, marketplace description, price, review score, and number of reviewers. This resulted in 482 apps, and after removing duplicates, 444 apps were included in the later selection.

The strategy for app selection outlined in Figure 1 aimed to include top-rated publicly available apps targeting primarily depression. From the initially identified 444 apps, we excluded those that (1) had less than 100 reviews; (2) were inaccessible at the time of selection; (3) belonged to irrelevant marketplace categories such as social, casual, business, news, or book; and (4) had average user review scores lower than 4.0 (out of 5.0). The application of these criteria on the initial set of 444 apps resulted in 94 apps for consideration.

From these apps, we further excluded those that did not focus primarily on depression by employing the following criteria: (1) the words “depression” or “depressed” do not appear in the app’s title or marketplace description of the app, (2) the primary target is not depression (eg, yoga tracker), and (3) their marketplace description mentions that people with depression should not use the app. These criteria led to 31 apps, from which we further excluded 2 more apps as their functionality was limited to the provision of therapy sessions to be purchased in-app. The remaining 29 apps were analyzed in this review (see Multimedia Appendix 1).

### Data Extraction

Descriptive characteristics of the apps were extracted from the information provided on the marketplace. These included *category, costs, target audience,* whether they claimed to be *evidence-based* (including explicit scientific underpinning and clinical input), and data supporting analysis of ethical aspects such as the *privacy policy*.

To extract data on app functionality, between June and October 2019, 2 rounds of experimental evaluation [36,37] were used in which the authors as HCI experts interacted with the apps using both Android and iPhone mobile devices (ie, Samsung tablet and Xiaomi phone for Android apps and iPhone for iOS apps). The entire set of apps was evaluated by 2 authors (CQ and CD), and 21% (6/29) of the apps were evaluated by all authors. The coding scheme was iteratively revised until agreement was reached among all the coders. The coding process was hybrid, integrating both deductive and inductive coding. Informed by prior work on the classification of mHealth apps [14], the deductive codes consisted of 3 main types of functionality of depression apps: screening, tracking, and provision of interventions (Table 1). The inductive coding [39] allowed the identification of specific subcodes under each of the main functionality described above. For instance, the screening function was broken down into subcodes such as symptom monitoring, self-diagnosis, and basis for personalization.

**Table 1:** Main codes and subcodes from functionality’s evaluation.

Functionality type and subtype		Definitions
**Screening**		
	Monitoring symptoms	The screening function is provided for monitoring depression symptoms during intervention
	Self-diagnosis	The screening function is provided for self-assessment of depression
	Basis for personalization	The screening function is provided as a basis for personalized intervention
**Tracking**		
	Tracking thought patterns	The tracking function supports the tracking of thought patterns
	Tracking mood patterns	The tracking function supports the tracking of users’ mood patterns
	Tracking behavior as the intervention progresses	The tracking function is provided for monitoring progress in following the intervention, including users’ adherence to the intervention
	Tracking depression symptoms	The tracking function is provided for monitoring symptoms
**Intervention**		
	Thought diaries	The intervention is provided to help users identify and challenge their negative thinking patterns
	Psychoeducation	The intervention is provided as psychoeducational content
	Mindfulness	The intervention is provided to help users improve mindfulness
	Behavioral techniques	The intervention is provided to motivate and guide users to perform positive behaviors
	Mood expression	The intervention is provided for users to express their emotions
	Other	The intervention is provided as emotional regulation strategies other than mindfulness

## Results

### Overview

The description of findings is organized into 3 parts. The first outlines a broader picture focusing on descriptive app characteristics (eg, categorization). The second part covers ethical considerations. The third part looks in more depth into specific functionality such as screening, tracking, and provision of interventions.

### Descriptive Characteristics

This section describes the characteristics of the selected apps, for example, the main categories under which depression apps are classified on marketplaces, their target audience, costs, evidence base, medical disclaimer, and whether involving of clinicians’ guidance while using the apps.

#### Categorization

The 29 apps reviewed in this study belong to 3 categories used to describe apps on the marketplaces. The most popular category is health and fitness (18/29, 62% apps), followed by lifestyle (4/29, 14% apps) and medical (7/29, 24% apps).

#### Targeted Audience (Age Group)

An important finding is that app marketplaces rate all apps as suitable for nonadult users (Multimedia Appendix 2). Most of the selected apps were classified as being suitable for children from preschool age: 76% (22/29) of apps were rated for those older than 3 years, 3% (1/29) for those older than 4 years, 7% (2/29) for those older than 12 years, 3% (1/29) for those older than 16 years, and 10% (3/29) with parental guidance.

However, only 41% (12/29) of the apps provide a privacy policy intended to protect children’s data. Half of these privacy policies (7/12, 58%) claim to restrict users to a specific age group, albeit this approach is inconsistent with the app’s age rating on the marketplace. For instance, one app (A8, see app_ID in Multimedia Appendix 2) states in its privacy policy that the app does not provide services to users who are younger than 18 years; in contrast, it is rated on the marketplace as Pan European Game Information (PEGI) 3. This may be because of a mismatch between age rating definitions oriented around the inclusion of material such as violent content, and health care apps that should have age restrictions because of the personal and sensitive nature of the content, with associated risk for harm.

In addition, all the apps apply the same design across all ages, and we did not find any customization for users who are children, such as involving in-app interactions to allow parents to collaborate or monitor their children while using the app [40].

#### Targeted Audience (Clinical Nosology)

All included apps claim to target users with depression. Most of the apps (20/29, 69%) represent *depression* as a lack of well-being (eg, feeling stressed or having low mood). Less than one-fifth of the apps (5/29, 17%) actually represent depression as a mental disorder, whereas only 1 app (A18) employs Patient Health Questionnaire-9 (PHQ-9) [41] to assess the severity of symptoms. Another 14% (4/29) of apps do not claim to target depression as a disorder, yet employ validated tools for assessing users’ depressive symptoms. Furthermore, none of the apps claims to target users with a specific level of severity (ie, mild, moderate, or severe depression).

#### Costs

An important finding is that although most of the apps (28/29, 97%) are free to download, at least some of their costs are covered either directly or indirectly by users (Multimedia Appendix 2). The direct costs consist of explicit charges for more advanced features, whereas indirect costs relate to users’ forced consumption of in-app advertisements. In-app purchase was offered by 66% (19/29) of the apps, mostly as a subscription priced between US $3.99 to US $29.99 per month, or as paid online therapy sessions (US $35/hourly session over call, video, or chat, A11). Advertisements were provided by 34% (10/29) of apps, which raises privacy concerns. Of the apps with advertisements, 80% (8/10) stated specifically in their privacy policies that users’ information, captured for instance through cookies, would be collected and shared with third parties, including advertisers or analytics providers. Only 1 app that offered advertisements claimed that users’ data would not be collected or shared (A29), whereas another app (A7) did not provide a privacy policy in English. Only 17% (5/29) of apps that are free to download neither request in-app purchase nor provide advertisement. Only 1 app requires purchase (for US $4.99) before downloading.

#### Evidence Base

Developers of 62% (18/29) of the apps have specified a scientific underpinning for their app design, whereas another 38% (11/29) do not make such a claim (Multimedia Appendix 3). Almost half of the apps (14/29, 48%) claim to be designed based on validated psychological treatments (eg, CBT, ACT, dialectical behavior therapy, and mindfulness). The remaining 14% (4/29) are designed based on theories pertaining to gamification, hypnosis, and affirmations. However, only 7% (2/29) of the apps provide direct evidence in the form of peer-reviewed scholarly work on the efficacy of the app for reducing depression symptoms [42,43], whereas another 34% (10/29) of apps provide indirect evidence of efficacy of their underpinning theories without referencing any academic work. For instance, 8 apps (A3, A4, A5, A15, A16, A17, A18, and A28) are promoted as evidence-based therapeutic tools by claims that their design is grounded on evidence-based treatments (ie, CBT). In addition, 41% (12/29) are described as being designed with input from clinicians (eg, psychologists, psychiatrists, and therapists), whereas 59% (17/29) do not mention the involvement of mental health professionals in their design.

#### Medical Disclaimer

A medical disclaimer is presented in 66% (19/29) of the apps, outlining that the app is not a replacement for clinical treatment (Multimedia Appendix 3). However, 11 out of these 19 apps (11/19, 58%) only present this disclaimer in their terms of use policy, which is difficult to find and unlikely to be read by users. Another 35% (10/29) of apps do not provide any disclaimer on either marketplace or app’s website. No app presented itself as an alternative to clinical treatment (ie, drug treatment or face-to-face psychotherapy).

#### Clinical Involvement

All apps are designed to be used independently and do not require professional guidance while using them (Multimedia Appendix 3). In addition, 5 apps (5/29, 17%) provide opportunities to involve health experts while using the app. Of these, 2 apps support access to coaching and counseling sessions as an additional intervention for a price ranging from US $29.99 per month (A27) to US $35 per hour (A11). The other 3 apps allow users to share their in-app data (eg, health tracking report) with their health care providers (A16, A22, and A24).

### Ethical Considerations

This section describes the ethical considerations raised while reviewing selected apps.

#### Negative Content

Aligned with the concerns raised by prior work that apps with poor design present an increased risk of potential harm [15,44], the results show that 2 out of 29 apps are categorized as so-called wallpaper apps. Such apps support people, “reflecting the true nature of the pain and loneliness in [your] heart […] give permission to feel the way you do” (A12). We found that these 2 apps include images or quotes capturing negative thinking (eg, “Do you ever get in those moods where you just don’t feel like existing,” A12). Surprisingly, these 2 apps with potentially disturbing content are rated as PEGI 3 (A12) or PEGI 12 (A6) on the marketplace, which indicates that the apps’ content merely includes bad language. As prior studies [45,46] have indicated, adolescents’ exposure to negative content may trigger negative behavior such as self-harm. Therefore, there is a clear need to explore safeguarding strategies for protecting vulnerable users such as those at risk of self-harm or suicide, especially given that these 2 apps are highly rated on the marketplace, ie, between 4.4 and 4.6 out of 5, and are subsequently more likely to be selected for use, adoption, or appropriation [47].

#### Safety

Strikingly, despite the increased vulnerability of people living with depression, 72% (21/29) of apps do not provide any information for handling or preventing the risk of suicide (Multimedia Appendix 4). Only 28% (8/29) of apps provide such information; in particular, most of these apps (5/8, 63%) provide information on accessing suicide prevention helplines, counseling websites, or support services, whereas 25% (2/8) provide information advising users to contact local emergency services if in critical risk of harm. In addition, 1 app (A18) assists users in creating a personalized safety plan for handling crises.

### Functionality Review

We now discuss the functionality of reviewed apps such as screening, tracking, and providing interventions.

#### Screening

A total of 9 apps offer functionality to screen for depression; their features are summarized in Multimedia Appendix 5. Almost half of the apps that provide screening functionality (4/9, 44%) aim to assess changes in users’ depression symptoms during engagement with the app-provided intervention. Interestingly, despite the acknowledged benefit of personalization to support adherence [48], most of these apps (3/4, 75%) provide predefined psychoeducation articles upon informing users of their screening result, rather than tailored information for addressing particular issues identified through screening. All 4 of these apps employ the PHQ-9, a validated screening tool. An interesting outcome in this context relates to the frequency of the screening. Although 2 apps supported periodic repeated measures of users’ depression (ie, apps suggest or limit access to the screening tool only once in a fortnight), another 2 apps instead allowed on-demand momentary screening of users’ depression (ie, users can access screening tools as frequently as they want with no instructions regarding an appropriate frequency).

In addition, 33% (3/9) of the apps provide stand-alone screening functionality for self-diagnosis purposes. Furthermore, 2 out of 3 apps classified into this category provide only screening functionality (A29 and A24), whereas another app (A16) also provides mood regulation strategies in addition to screening as its primary function. The first 2 apps (A29 and A24) do not use validated screening tools and do not provide direct in-app links to professional help upon informing users of the severity of their screening results. We found that the other app (A16) enables the potential benefits of screening while avoiding harm, as it provides support for both psychoeducation and for discussing the diagnosis and its implications with mHealth professionals [15,19]. In addition, the app (A16) provides screening as the main functionality through the use of International Classification of Diseases-10 [49], a validated screening tool, and in-app links to professional support. A16 also allows users to generate a report of the screening result to show to their own health care professionals.

The other apps (2/9, 22%) provide a screening function to inform the delivery of personalized app content. One app asks users to self-report their disorder and symptoms (A19), whereas another app uses a questionnaire as a screening tool (A11), although it provides neither the source of this questionnaire and information on its validity nor evidence for the personalization of intervention. This app offers in-app purchase of online therapy sessions; however, this is not integrated with users’ progress through the intervention or their screening results.

#### Tracking

Out of the 29 apps, 19 apps offer functionality for tracking at least one aspect such as *thoughts*, *behaviors, moods*, or *depression symptoms* (Multimedia Appendix 6).

Apps that track multiple aspects serve different purposes; 89% (17/19) of these apps support tracking to assist the provision of personalized intervention, ie, tracking *thought* changes for providing materials to apply within the intervention or tracking users’ *behavior* for visualizing their progress and adherence to the intervention. Furthermore, 37% (7/19) of the apps support *mood* tracking for revealing their triggers and patterns. Another 26% (5/19) of apps support tracking of *symptoms of depression* through frequent use of screening tools, and 1 of these 5 apps (A16) tracks aspects such as thought changes, mood, or physical condition (ie, appetite, sleep) over fortnightly periods to generate the screening result.

Thought tracking is supported by 74% (14/19) of the tracking apps, mostly combined with mood tracking on the same data entry. Good practices for improving usability have started to emerge, for instance, in the form of templates for guiding users through the tracking process (available in 11/14, 79% apps). There is also an opportunity to explore alternative modalities for mood tracking. From the selected apps, we found that text is the most commonly employed modality for recording thoughts (14/14, 100% apps) and moods (9/14, 64% apps). Other modalities such as emoticons are being used to record moods tagged with thoughts (4/9, 44%), and scales are being used to record mood intensity (1/9, 11%). Opportunities also arise for better representing the thought logs, for instance, introducing searching or filtering functionality. Currently, all 14 apps present thought logs directly to users in chronological order without the option of searching them.

Of the 42% (8/19) apps that track user *behavior* as progress through the intervention, 3 apps automatically log users’ adherence to the proposed usage goals for app-delivered intervention (eg, minutes spent on app-delivered meditation), whereas 5 apps track user’s achievement of positive behaviors suggested by the app (eg, socializing with friends and drinking water). Apps for the latter purpose mostly require users to log their achieved activity themselves, whereas 1 app allows automatic tracking (ie, step count, A13). In addition, only half of the progress-tracking apps (5/8, 63%) provide a summary visualization of intervention progress (2 apps provide a graphical summary, eg, A11 provides a calendar view). Another 3 apps provide a textual summary (eg, A17 displays the total number of minutes of meditation, without providing a record of each specific meditation). The other 38% (3/8) of apps provide direct access to textual logs with no summary.

In addition, 37% (7/19) of the apps support the understanding of *mood* patterns through visualizations. Such apps often track moods alongside their triggering factors (available in 4 apps) or physical conditions such as headache (available in 4 apps); the aim of the former is to understand the reasons for changes in mood, whereas the latter aims to reveal the impact of physical conditions on such changes. Despite the clear purpose of supporting understanding articulated by developers, the representation of logged data does not easily support the understanding of data patterns. Even though a graphical view of mood changes over time is provided by all 7 apps, most of them (4/7, 57%) provide it separately from the graphical view of other tracked factors (eg, A14, A28, and A11 provide a graphical view of mood changes within a period and a textual representation of mood triggering factors). Another 3 apps (3/7, 43%) offer an integrated representation of changes in physical condition with changes in mood, which may make it easier to understand relationships between the two.

Furthermore, 26% (5/19) of the apps automatically track screening results for *symptom* monitoring. Most of these apps (4/5) provide only a textual review of screening results, in chronological order. Only 1 app (A28) also provides a graphic visualization of changes in screening results.

#### Interventions

Overall, 5 types of interventions were identified in the analysis (see Multimedia Appendix 7), reflecting a mixture of elements from psychological interventions, including *thought diaries, psychoeducation, mindfulness, scheduling positive behaviors,* and *others.* A distinct group of apps aims to support *emotional expression* rather than a particular psychological intervention.

Thought diaries are a common intervention employed by one-third of the apps (9/24, 38%). This intervention borrows from traditional CBT practice by providing instructions for identifying negative thought patterns and for challenging distorted thoughts. One approach to tailoring interventions is to employ guidance for challenging real-time tracked thoughts or emotions. Most of these apps (7/9, 78%) provide thought diaries as tailored interventions consisting of guidance for identifying and selecting personal challenging thought patterns to guide the writing of reflective diaries. Another 2 apps provide a generic template to guide thought diaries, rather than adaptive or personalized guidance.

Apart from thought dairies, another set of 9 apps (9/24, 38%) provide specific *psychoeducation* as an intervention. Findings suggest that 44% (4/9) of such content is provided to specifically fit users’ depression assessment, whereas 56% (5/9) is nonpersonalized, generic content.

Mindfulness [50] is another popular intervention (11/24, 46%) as most of the selected apps include meditation (9 apps), grounding techniques (1 app, A26), or breathing guides (1 app, A2). Furthermore, 4 apps suggest a frequency of use for the intervention, eg, 1 meditation session per day (A1), whereas the others do not specify a frequency of use. In addition, 2 apps provide adaptive interventions (ie, meditation guidance) triggered by users’ input (eg, during users’ conversation with artificial intelligence [AI]–based chatbot, A27 and A28).

In addition, 17% (4/24) of the apps delivered interventions for *scheduling positive behaviors* (or behavior activation). Aligned with prior work, personalization [19,29] is a good design principle for engaging users with app-delivered interventions. Overall, 3 apps offer tailored intervention materials by allowing users to enter positive behaviors that they wish to schedule (eg, A15, A18, and A21), and another app (A11) provides a personalized monthly plan based on the results of the users screening measures. Other valuable design choices supporting engagement include offering peer support [19] during the intervention (1 app, A21) or using gamification for providing daily intervention goals and rewards [51] for completed activities (2 apps, A11 and A21).

A final category of apps is those helping users to *express their emotions* associated with depression (5/24, 21), either by sharing posts in online support groups or by individually consuming art-based materials. Of the 2 apps providing peer-supported mood expression, only 1 provides links to a 24/7 suicide helpline. Both apps allow users to filter posts: 1 app (A23) allows users to set filter words (eg, “suicide”) to hide posts including such words and safeguard themselves from such content, whereas another app (A19) filters materials (ie, posts in the community) automatically and only shows materials that relate to users’ self-reported disorder and symptoms. Apps that fall in the latter category (3/5, 60%) provide art-based content for expressing depressive moods, eg, wallpaper pictures with emotional quotes. However, an important concern is that none of the wallpaper apps provide any scientific background or features to support access to mental health services for users at risk of suicide or self-harm. Most of the content of these 3 apps are negative, and only 1 of these apps also provides some positive content, being also the only app that offers users the possibility of personalizing the quotes.

Another 3 apps provide *other* types of emotion regulation strategies, including positive affirmations (1 app, A25) or hypnosis (2 apps, A10 and A20). Customization of intervention material is available in 1 app (A25), which allows users to create positive affirmations and to audio record them.

## Discussion

### Principal Findings

This paper indicates that the current top-ranked apps for depression provide various features to benefit users across different age groups. The potential of this newly established marketplace is promising, especially for reaching subgroups of users such as adolescents, who are less likely to seek professional support offline and thus could benefit from appropriately designed mHealth apps. For this purpose, we discuss the need and opportunity for regulating the marketplace to safeguard users and to ensure a positive impact from the use of apps.

We begin by considering the ethical principle of nonmaleficence [52] within the top-rated apps for depression. First, a clearer definition of age restrictions on the marketplace could better support users in general and younger users in particular to select age-appropriate apps. We found age to be handled insufficiently and inconsistently in current commercial apps, given that the age ratings on the marketplace generally indicate the maturity of app content rather than the targeted users for the app, and we also found that these ratings were generally inconsistent with information regarding the targeted age group. This risk is further heightened by the conditions within the reviewed apps’ privacy policies including the sharing of users’ data with third parties for commercial purposes.

A recent systematic review of HCI work on affective health technologies also identified potentially harmful aspects of tracking apps such as the provision of negative mood or thinking patterns with insufficient professional support, inadequate screening, and insufficiently founded diagnosis claims based on tracked data [30]. With respect to communicating negative content, we see apps supporting the consumption of publicly shared emotional expressions of depression generated by others (A6 and A12). We further advocate that developers should consider the presence of negative content when selecting an age rating on the marketplace, as consumption of such content may lead to harmful behavior among adolescent users.

In addition, this paper systematically reviewed and analyzed the apps’ functionality. The result inspires recommendations to guide developers to further leverage digital affordances to mitigate harm, to deliver personalized depression treatments, and to track multimodal content. For instance, for apps that provide screening functionality, there may be a tendency to overclaim symptom screening informed by nonvalidated screening tools rather than using validated ones, eg, developers of A24 and A29 prominently state their apps’ effectiveness in clinical practice on the marketplace but do not provide scientific validation for the screening tools employed. In addition, with regard to the increased vulnerability of depressed individuals, we find limited direct access to professional help when screening results are communicated to users. For instance, in general, 76% (22/29) apps do not provide immediate access to suicide prevention or online counseling helplines (Multimedia Appendix 3).

### Safeguarding Users While Accessing and Consuming Negative Content

Risk of harm can be identified with respect to the viewing of strongly negative content from others within the emotional expression apps for depression. Our findings highlight strong ethical concerns around these apps. Although arguably beneficial for people creating it [53], such content might have a negative effect on those viewing it, especially given that depressed individuals have a tendency toward rumination [54]. We suggest that such apps should include safeguards for users viewing highly negative content. Moreover, developers of such apps could limit views of negative content, especially given that these 2 apps (A6 and A12) are also accessible to adolescent users, who are susceptible to engage in *problem* or *at-risk* behaviors [40]. One deployed strategy was to automatically cover negative keywords within app-provided content and to offer a pop-up window with free psychological counseling helpline every 3 times when users choose to reveal the hidden negative words (A23).

In addition, apps not specifically designed for children and adolescents, but with a child-friendly age rating on the marketplace, should consider introducing customizable designs for nonadult users. It has previously been suggested that providing support and treatment sessions with parents, teachers, and siblings should be seriously considered when administering treatment to children with depression [40]. Therefore, we suggest that designers of such apps should consider mechanisms to engage parental support or supervision while children or adolescents are using these apps.

An interesting issue with respect to apps supporting the tracking of mood and thought patterns is the unfiltered presentation of these data when predominantly negative content is being tracked. Apps tracking thoughts only provide access to tracking logs in chronological order, and this presents a 2-fold limitation. First, such visualizations can be browsed but not queried to retrieve a specific entry. Second, browsing such logs may trigger vivid recall when they capture negative content and may increase the risk of rumination [29].

### Safeguarding Users While Selecting Age-Appropriate Apps and Sharing Private Data

The suggestions discussed in this section particularly target the developers of marketplaces hosting apps for depression. Previous findings suggested that the regulation of such apps regarding data privacy remains inadequate [25,32,35] and reported the prevalence of health-related apps selling users’ data to third parties. Survey studies have also indicated that the general public is less inclined to share their health care data with technology companies [32]. The identified limitations of the privacy policies for the reviewed apps illustrate that these concerns can be better addressed; 24% (7/29) of the apps failed to provide any privacy policy in English or in a reliable source (Multimedia Appendix 2). In addition, aligned with prior studies [26,55], the current privacy policies may be difficult to comprehend by typical users. We, thus, call for developers to improve the readability of privacy policies and support the suggestion of making them easy to read at a sixth-grade reading level [26].

Another concern is protecting the privacy of users’ health data and, in particular, the data of young people while using depression apps. First, more than half of these apps (24/29, 83%) fail to provide privacy policies that specify strategies to protect children’s data (16/29, 55%). Second, our findings also show that although most of the apps are free to download, they normally come with in-app purchases for additional features or advertisements. Regarding advertisement, we found that 80% (8/10) of apps that use advertisements declare that they share users’ data for commercial purposes.

All of the reviewed apps are rated as suitable for children and adolescents on the marketplace, whereas one-fifth (7/29, 24%) of the apps specifically claim to restrict access from young users. This finding demonstrates the need for developers of marketplaces that host depression apps to increase the transparency of their standards. For instance, Google specifies that [56] their age rating is not for describing the apps’ target user group but rather for describing the minimum maturity level of content in apps such as violence, drugs, and profane language.

Surprisingly, however, no statement regarding data sharing or targeted users’ age range could be found on the app descriptions in the marketplace to support users making an informed decision at the point of downloading the app. The age rating may be specifically misleading to parents when they are selecting age-appropriate apps for their children as developers only claim age restrictions in the privacy policy. We advocate a clearer definition and regulations for age rating of depression apps on marketplaces.

In addition, we argue that users should be informed upfront of the risk of having their sensitive data shared with third parties for commercial purposes. The prevalence of health-related apps selling users’ data to third parties has been previously reported [25,35,57]. Thus, we argue for the responsibility on the marketplaces’ developers to ensure consistency of privacy-related information in the app description on the marketplaces when compared with its privacy policy or to ensure that the privacy policy is included directly within the app.

### Safeguarding Users While Screening for Depression

Prior studies [57] have reported the tendency of commercial depression apps to blur the line between depression as a lack of wellness or as a mental disorder, which aligns with our findings. In addition, none of the apps examined claim to target a specific level of depression severity. Although apps may potentially reach a wider range of users by following such a strategy, it may be more difficult to formulate appropriate safeguards for users whose depression leaves them with higher levels of vulnerability [57]. In addition, we found that most depression apps tend not to undergo a rigorous evaluation of their intervention components but instead rely on designing the app based on evidence-based theory [26]. Apps with insufficient evidence of efficacy present challenges as they may risk misinforming patients [57]. We advocate clear communication of the targeted user groups for mHealth apps and marketplace guidelines to match the required level of evidence for each app as well as the condition and risks of their specifically targeted user group.

App-based depression assessment is potentially valuable in supporting individuals with depression concerns to seek help and share their electronic health information with health professionals [15,26]. In addition, health data collected by users could support professionals’ understanding of users’ symptoms, which could support diagnosis and the delivery of clinical treatment. Despite these potential benefits, the top-rated depression apps reviewed seldom support this usage. Only 1 of 8 apps offered the option of generating reports of screening outcomes for sharing with mental health professionals.

Although PHQ-9 is the most used tool for depression screening, 3 out of 8 apps use nonvalidated screening tools, and information about screening tools and their scientific underpinning is seldom provided within app descriptions. We recommend that app developers use validated screening tools and provide basic information about the tools and their validity.

In addition, findings indicate that screening tools employing periodic repeated measures such as PHQ-9 [41] also tend to be used within apps during daily tracking. However, the latter may be better suited to more lightweight ecological momentary assessment measures [58] rather than depression diagnosis measures. We also found a few emerging practices addressing this concern by suggesting an appropriate frequency for screening or even limiting the frequency of access to screening tools (A16 and A28). Thus, we suggest that app developers decouple the use of periodically repeated measures such as PHQ-9 for the purpose of depression screening and the use of ecological momentary assessment for more frequent daily tracking of mood, thoughts, behavior patterns, and symptoms of depression [59].

### Opportunity to Improve Apps for Depression by Leveraging Digital Affordances

An important challenge of mobile apps for depression is attrition [29,60]. Previous work suggested the value of personalization for improving users’ engagement with apps [19,29,61] and the value of accessing social support [19] and involving concepts from gamification [51]. In the future, this may involve the provision of real-time adaptive personalization of intervention content to the tracked thoughts or emotions [59]. However, despite the potential of mobile technology to deliver personalization, apps supporting it are limited. Exceptions here include the use of AI chatbot conversational agents (A2 and A28) to respond in real time to users’ currently recorded thoughts, instead of generic (not personalized) psychoeducational content. Personalization can also be extended to the schedule of activities within an app-delivered intervention. However, only 1 of the reviewed apps (A11) offered a personalized intervention plan based on users’ screening results. There is an opportunity to better leverage digital affordances for personalization when designing apps for depression.

Findings also indicate that tracking within depression apps is focused on capturing users’ mood patterns or thought patterns and their engagement with app-delivered interventions. However, these distinct types of tracked content are seldom available together in a single app. We argue for the value of simultaneously capturing both thinking and emotional content as these can support better encoding at the moment when an event occurs and better retrieval later [62,63]. We also suggest that integrating such tracked content with a record of progress through the intervention and completion of intervention activities could better allow users to understand the value of the app for their well-being. Such combined visualization could further support users’ engagement and motivation to continue to use the app-delivered intervention.

### Conclusions and Future Work

The rapid increase of mobile apps for reducing depression can benefit from a closer look and evaluation of the functionality such apps actually deliver and the potential ethical issues that they raise. From a systematic analysis of 29 top-rated depression apps on the major marketplaces, we suggest that developers of marketplaces should regulate depression apps to mitigate ethical risks, including missing, inadequate, or inconsistent privacy policies, ie, sharing data with third parties, child data protection, and safeguarding of vulnerable user groups. In addition, the analysis of app functionality provided new insights into opportunities for mitigating harm regarding the consumption of the negative content, unrestricted access by children (with related privacy concerns), and the provision of screening tools with less scientific validation.

## Appendix

Multimedia Appendix 1 List of selected apps.

Multimedia Appendix 2 Assessment of targeted audience and costs.

Multimedia Appendix 3 Assessment of apps’ evidence base.

Multimedia Appendix 4 Assessment of Safety design.

Multimedia Appendix 5 Assessment of the screening functionality (9 apps).

Multimedia Appendix 6 Assessment of the tracking functionality (19 apps).

Multimedia Appendix 7 Assessment of the intervention functionality (24 apps).

## Abbreviations

ACT: acceptance and commitment therapy
AI: artificial intelligence
CBT: cognitive behavior therapy
HCI: human-computer interaction
iOS: iPhone OS
mHealth: mobile health
PEGI: Pan European Game Information
