# Correction: Functionality of Top-Rated Mobile Apps for Depression: Systematic Search and Evaluation

**DOI:** 10.2196/18042

**Published:** 2020-02-21

**Authors:** Chengcheng Qu, Corina Sas, Claudia Daudén Roquet, Gavin Doherty

**Affiliations:** 1 School of Computing and Communications Lancaster University Lancaster United Kingdom; 2 School of Computer Science and Statistics Trinity College Dublin Dublin Ireland

The authors of “Functionality of Top-Rated Mobile Apps for Depression: Systematic Search and Evaluation” (JMIR Mental Health 2020;7(1):e15321) noticed two errors in their published manuscript.

The Acknowledgments section was omitted from the end of the paper. It should read:

This work has been supported by AffecTech: Personal Technologies for Affective Health, Innovative Training Network funded by the H2020 People Programme under Marie Sklodowska-Curie grant agreement number 722022. The research of GD is funded in part by SFI grant number 13/RC/2106 to the Adapt Centre.

Additionally, in the Discussion section, under the subheading “Conclusions and Future Work”, there was an extra word (“employing”) in the following sentence:

In addition, the analysis of app functionality provided new insights into opportunities for mitigating harm regarding the consumption of the negative content, unrestricted access by children (with related privacy concerns), and the provision of screening employing tools with less scientific validation.

The corrected sentence is:

In addition, the analysis of app functionality provided new insights into opportunities for mitigating harm regarding the consumption of the negative content, unrestricted access by children (with related privacy concerns), and the provision of screening tools with less scientific validation.

The corrections will appear in the online version of the paper on the JMIR website on February 21, 2020, together with the publication of this correction notice. Because this was made after submission to PubMed, PubMed Central, and other full-text repositories, the corrected article has also been resubmitted to those repositories.

