# Supplementary material for: Functionality of Top-Rated Mobile Apps for Depression: Systematic Search and Evaluation
Source: JMIR Ment Health. 2020 Jan 24;7(1):e15321. doi: 10.2196/15321 (PMC7007593; doi:10.2196/15321)
Supplement: Multimedia Appendix 1 [file mental_v7i1e15321_app1.docx]

| App_ID | App_name | App_Link |
| --- | --- | --- |
| 1 | Aware: Meditation & Mindfulness | [https://play.google.com/store/apps/details?id=com.mindfulness.aware&hl=en. Accessed: 2019-06-06.](https://play.google.com/store/apps/details?id=com.mindfulness.aware&hl=en.%20Accessed:%202019-06-06.) |
| 2 | Breathe Easy | [https://play.google.com/store/apps/details?id=com.moodtools.breatheeasy&hl=en_US. Accessed: 2019-06-24.](https://play.google.com/store/apps/details?id=com.moodtools.breatheeasy&hl=en_US.%20Accessed:%202019-06-24.) |
| 3 | CBT Thought Record Diary | [https://play.google.com/store/apps/details?id=com.moodtools.cbtassistant.app. Accessed: 2019-06-06.](https://play.google.com/store/apps/details?id=com.moodtools.cbtassistant.app.%20Accessed:%202019-06-06.) |
| 4 | Cognitive Diary CBT Self-Help | [https://play.google.com/store/apps/details?id=com.excelatlife.cbtdiary&hl=en_GB. Accessed: 2019-06-06.](https://play.google.com/store/apps/details?id=com.excelatlife.cbtdiary&hl=en_GB.%20Accessed:%202019-06-06.) |
| 5 | Depression CBT Self-Help Guide | [https://play.google.com/store/apps/details?id=com.excelatlife.depression. Accessed: 2019-06-06.](https://play.google.com/store/apps/details?id=com.excelatlife.depression.%20Accessed:%202019-06-06.) |
| 6 | Depressive and sad wallpaper | [https://play.google.com/store/apps/details?id=com.sadanddpressivewallpapers&hl=gb. Accessed: 2019-07-01.](https://play.google.com/store/apps/details?id=com.sadanddpressivewallpapers&hl=gb.%20Accessed:%202019-07-01.) |
| 7 | Disappointment Quotes | [https://play.google.com/store/apps/details?id=com.khoniadev.frasesdedecepcion&hl=gb. Accessed: 2019-07-01.](https://play.google.com/store/apps/details?id=com.khoniadev.frasesdedecepcion&hl=gb.%20Accessed:%202019-07-01.) |
| 8 | eMoods Bipolar Mood Tracker | [https://play.google.com/store/apps/details?id=my.tracker&hl=en. Accessed: 2019-06-06.](https://play.google.com/store/apps/details?id=my.tracker&hl=en.%20Accessed:%202019-06-06.) |
| 9 | Fight Depression Naturally | [https://play.google.com/store/apps/details?id=com.andromo.dev462136.app429809. Accessed: 2019-06-24.](https://play.google.com/store/apps/details?id=com.andromo.dev462136.app429809.%20Accessed:%202019-06-24.) |
| 10 | Hypnosis for Anxiety, Stress Relief & Depression | [https://play.google.com/store/apps/details?id=com.pitashi.audiojoy.empoweredhypnosisanxiety&hl=en_GB. Accessed: 2019-06-24.](https://play.google.com/store/apps/details?id=com.pitashi.audiojoy.empoweredhypnosisanxiety&hl=en_GB.%20Accessed:%202019-06-24.) |
| 11 | InnerHour - Self Help for Anxiety & Depression | [https://play.google.com/store/apps/details?id=com.theinnerhour.b2b&hl=en_GB. Accessed: 2019-06-06.](https://play.google.com/store/apps/details?id=com.theinnerhour.b2b&hl=en_GB.%20Accessed:%202019-06-06.) |
| 12 | Lonely Wallpaper | [https://play.google.com/store/apps/details?id=com.altland.lonely&hl=en_GB. Accessed: 2019-06-27.](https://play.google.com/store/apps/details?id=com.altland.lonely&hl=en_GB.%20Accessed:%202019-06-27.) |
| 13 | MindCare: mental well-being analytics made easy | [https://play.google.com/store/apps/details?id=com.blue_bird_tech.mindcare&hl=en_GB. Accessed: 2019-06-17.](https://play.google.com/store/apps/details?id=com.blue_bird_tech.mindcare&hl=en_GB.%20Accessed:%202019-06-17.) |
| 14 | Mood Log | https://play.google.com/store/apps/details?id=arproductions.andrew.moodlog&hl=en. Accessed: 2019-06-06. |
| 15 | MoodKit - Mood Improvement Tools | <https://itunes.apple.com/us/app/vertigo-music-share-life/id1118149022.> |
| 16 | Moodpath - Depression & Anxiety Test | [https://play.google.com/store/apps/details?id=de.moodpath.android&hl=en_GB. Accessed: 2019-06-03.](https://play.google.com/store/apps/details?id=de.moodpath.android&hl=en_GB.%20Accessed:%202019-06-03.) |
| 17 | MoodSpace | [https://play.google.com/store/apps/details?id=boundless.moodgym&hl=en_GB. Accessed: 2019-06-06.](https://play.google.com/store/apps/details?id=boundless.moodgym&hl=en_GB.%20Accessed:%202019-06-06.) |
| 18 | MoodTools - Depression Aid | [https://play.google.com/store/apps/details?id=com.moodtools.moodtools. Accessed: 2019-06-03.](https://play.google.com/store/apps/details?id=com.moodtools.moodtools.%20Accessed:%202019-06-03.) |
| 19 | We are more - our support network (Original name: Reachout: My Support Network) | [https://play.google.com/store/apps/details?id=com.reachout&gl=GB. Accessed: 2019-10-16.](https://play.google.com/store/apps/details?id=com.reachout&gl=GB.%20Accessed:%202019-10-16.) |
| 20 | Relieve Depression Hypnosis - Mood & Anxiety Help | https://play.google.com/store/apps/details?id=com.surfcityapps.relievedepression&hl=en_GB. Accessed: 2019-06-24. |
| 21 | SuperBetter | [https://play.google.com/store/apps/details?id=com.superbetter.paid&hl=en_GB. Accessed: 2019-06-06.](https://play.google.com/store/apps/details?id=com.superbetter.paid&hl=en_GB.%20Accessed:%202019-06-06.) |
| 22 | T2 Mood Tracker | https://play.google.com/store/apps/details?id=com.t2.vas. Accessed: 2019-06-17. |
| 23 | TalkLife | [https://talklife.co/. Accessed: 2019-06-28.](https://talklife.co/.%20Accessed:%202019-06-28.) |
| 24 | The Szondi Test: Research of Depression | [https://play.google.com/store/apps/details?id=lenin.szondi.test&hl=en_GB. Accessed: 2019-06-03.](https://play.google.com/store/apps/details?id=lenin.szondi.test&hl=en_GB.%20Accessed:%202019-06-03.) |
| 25 | ThinkUp: Positive Affirmations | [https://play.google.com/store/apps/details?id=com.think.up&hl=en_GB. Accessed: 2019-06-24.](https://play.google.com/store/apps/details?id=com.think.up&hl=en_GB.%20Accessed:%202019-06-24.) |
| 26 | What's Up? - Mental Health App | [https://play.google.com/store/apps/details?id=com.jacksontempra.apps.whatsup. Accessed: 2019-06-06.](https://play.google.com/store/apps/details?id=com.jacksontempra.apps.whatsup.%20Accessed:%202019-06-06.) |
| 27 | Wysa: stress, depression & anxiety therapy chatbot | [https://play.google.com/store/apps/details?id=bot.touchkin. Accessed: 2019-06-06.](https://play.google.com/store/apps/details?id=bot.touchkin.%20Accessed:%202019-06-06.) |
| 28 | Youper - Anxiety & Depression | [https://play.google.com/store/apps/details?id=br.com.youper&hl=en_GB. Accessed: 2019-06-03.](https://play.google.com/store/apps/details?id=br.com.youper&hl=en_GB.%20Accessed:%202019-06-03.) |
| 29 | 🇬🇧Depression Test | [https://play.google.com/store/apps/details?id=com.programming.advanced.depressiontest&hl=en_GB. Accessed: 2019-06-17.](https://play.google.com/store/apps/details?id=com.programming.advanced.depressiontest&hl=en_GB.%20Accessed:%202019-06-17.) |
| 7 | Disappointment Quotes | [https://play.google.com/store/apps/details?id=com.khoniadev.frasesdedecepcion&hl=gb. Accessed: 2019-07-01.](https://play.google.com/store/apps/details?id=com.khoniadev.frasesdedecepcion&hl=gb.%20Accessed:%202019-07-01.) |
| 8 | eMoods Bipolar Mood Tracker | [https://play.google.com/store/apps/details?id=my.tracker&hl=en. Accessed: 2019-06-06.](https://play.google.com/store/apps/details?id=my.tracker&hl=en.%20Accessed:%202019-06-06.) |
| 9 | Fight Depression Naturally | [https://play.google.com/store/apps/details?id=com.andromo.dev462136.app429809. Accessed: 2019-06-24.](https://play.google.com/store/apps/details?id=com.andromo.dev462136.app429809.%20Accessed:%202019-06-24.) |
