# Supplementary material for: Functionality of Top-Rated Mobile Apps for Depression: Systematic Search and Evaluation
Source: JMIR Ment Health. 2020 Jan 24;7(1):e15321. doi: 10.2196/15321 (PMC7007593; doi:10.2196/15321)
Supplement: Multimedia Appendix 2 [file mental_v7i1e15321_app2.docx]

| App_ID | App_name | Age rating (marketplace) | Any costs (marketplace)? | Child protection (privacy policy) | Do they have a privacy policy? | Of the apps with ads, do they claim to share data with 3rd parties? |
| --- | --- | --- | --- | --- | --- | --- |
|  |  |  |  |  |  |  |
| A1 | Aware: Meditation & Mindfulness | PEGI 3 | Offers in-app purchases | N/A | Yes |  |
| A2 | Breathe Easy | PEGI 3 | N/A | N/A | Yes, but in a less reliable source (available as a medium page) |  |
| A3 | CBT Thought Record Diary | PEGI 3 | Offers in-app purchases | N/A | Yes, but in a less reliable source (available as a medium page) |  |
| A4 | Cognitive Diary CBT Self-Help | PEGI 3 | Offers in-app purchases, contains ads | Do not collect data from users younger than 13 years, unless with parents' consent | Yes | Share data with 3rd parties. E.g., advertisers |
| A5 | Depression CBT Self-Help Guide | PEGI 3 | Offers in-app purchases, contains ads | Do not collect data from users younger than 13 years, unless with parents' consent | Yes | Share data with 3rd parties. E.g., advertisers |
| A6 | Depressive and sad wallpaper | PEGI 12 | Contains ads | N/A | Yes | Share data with 3rd parties. E.g., advertisers |
| A7 | Disappointment Quotes | PEGI 16 | Contains ads | N/A | Yes but not in English | Data privacy not in English |
| A8 | eMoods Bipolar Mood Tracker | PEGI 3 | Offers in-app purchases | Only provide service to users older than 18 years | Yes |  |
| A9 | Fight Depression Naturally | PEGI 3 | Offers in-app purchases, Contains ads | N/A | Yes | Share data with 3rd parties. E.g., advertisers |
| A10 | Hypnosis for Anxiety, Stress Relief & Depression | PEGI 3 | Offers in-app purchases | Do not collect data from users younger than 13 years | Yes |  |
| A11 | InnerHour - Self Help for Anxiety & Depression | PEGI 3 | Offers in-app purchases | Only provide service to users older than 18 years | Yes |  |
| A12 | Lonely Wallpaper | PEGI 3 | Contains ads | Only provide service to users older than 13 years | Yes | Share data with 3rd parties |
| A13 | MindCare: mental well-being analytics made easy | PEGI 3 | N/A | N/A | Yes, but in a less reliable source (available as a shared dropbox document), and not in English |  |
| A14 | Mood Log | PEGI 3 | N/A | Only provide service to users older than 13 years | Yes |  |
| A15 | MoodKit - Mood Improvement Tools | 12+ | $4.99 | N/A | Yes |  |
| A16 | Moodpath - Depression & Anxiety Test | PEGI 3 | Offers in-app purchases | N/A | Yes |  |
| A17 | MoodSpace | PEGI 3 | Offers in-app purchases | N/A | Yes |  |
| A18 | MoodTools - Depression Aid | PEGI 3 | Offers in-app purchases | N/A | Yes, but in a less reliable source (available as a medium page) |  |
| A19 | We are more - our support network | parental guidance | Offers in-app purchases, Contains ads | Do not collect data from users younger than 18 | Yes | Share data with 3rd parties. E.g., advertisers |
| A20 | Relieve Depression Hypnosis - Mood & Anxiety Help | PEGI 3 | Offers in-app purchases, contains ads | Only provide service to users older than 13 | Yes | Share data with 3rd parties. E.g., advertisers |
| A21 | SuperBetter | parental guidance | N/A | Do not collect data from users younger than 13 | yes |  |
| A22 | T2 Mood Tracker | PEGI 3 | N/A | N/A | No |  |
| A23 | TalkLife | parental guidance | Offers in-app purchases, contains ads | N/A | Yes | Share data with 3rd parties. E.g., advertisers |
| A24 | The Szondi Test: Research of Depression | PEGI 3 | Offers in-app purchases | N/A | Yes, but in a less reliable source (available as a shared google drive document) |  |
| A25 | ThinkUp: Positive Affirmations | 4+ | Offers in-app purchases | Only provide service to users older than 18; or users between 14 and 18 years and use the app with parental guidance | Yes |  |
| A26 | What's Up? - Mental Health App | PEGI 3 | Offers in-app purchases | N/A | No |  |
| A27 | Wysa: stress, depression & anxiety therapy chatbot | PEGI 3 | Offers in-app purchases | Only provide service to users older than 13 years | Yes |  |
| A28 | unless - Anxiety & Depression | PEGI 3 | Offers in-app purchases | N/A | Yes |  |
| A29 | 🇬🇧Depression Test | PEGI 3 | Contains ads | N/A | Yes, but in a less reliable source (available as a shared google drive document) | No data is collected or shared |
