# Supplementary material for: Functionality of Top-Rated Mobile Apps for Depression: Systematic Search and Evaluation
Source: JMIR Ment Health. 2020 Jan 24;7(1):e15321. doi: 10.2196/15321 (PMC7007593; doi:10.2196/15321)
Supplement: Multimedia Appendix 3 [file mental_v7i1e15321_app3.docx]

| App_ID | App_name | Claimed scientific underpinning | Claimed evidence base | Clinical input during development | Medical disclaimer | Clinical involvement while using the app |
| --- | --- | --- | --- | --- | --- | --- |
|  |  |  |  |  |  |  |
| A1 | Aware: Meditation & Mindfulness | mindfulness | (Indirect evidence) Claimed effectiveness of background science | - | Yes but hard to find | - |
| A2 | Breathe Easy | - | - | - | N/A | - |
| A3 | CBT Thought Record Diary | CBT | (Indirect evidence) Claimed effectiveness of background science | - | N/A | - |
| A4 | Cognitive Diary CBT Self-Help | CBT | (Indirect evidence) Claimed effectiveness of background science | - | Yes | - |
| A5 | Depression CBT Self-Help Guide | CBT | (Indirect evidence) Claimed effectiveness of background science | - | Yes but hard to find | - |
| A6 | Depressive and sad wallpaper | - | - | - | N/A | - |
| A7 | Disappointment Quotes | - | - | - | N/A | - |
| A8 | eMoods Bipolar Mood Tracker | CBT | - | - | Yes | - |
| A9 | Fight Depression Naturally | - | - | - | Yes | - |
| A10 | Hypnosis for Anxiety, Stress Relief & Depression | Hypnosis | - | ✓ | Yes but hard to find | - |
| A11 | InnerHour - Self Help for Anxiety & Depression | CBT, Positive Psychology, Mindfulness | - | ✓ | Yes but hard to find | Users can activate standby coaches or therapists in app. |
| A12 | Lonely Wallpaper | - | - | - | N/A | - |
| A13 | MindCare: mental well-being analytics made easy | CBT, DBT | - | - | Yes | - |
| A14 | Mood Log | - | - | - | N/A | - |
| A15 | MoodKit - Mood Improvement Tools | CBT | (Indirect evidence) Claimed effectiveness of background science | ✓ | Yes but hard to find | - |
| A16 | Moodpath - Depression & Anxiety Test | CBT | (Indirect evidence) Claimed effectiveness of background science | ✓ | Yes but hard to find | Users can generate report to show clinicians |
| A17 | MoodSpace | CBT, Mindfulness based cognitive therapy (mbct), Positive psychology, Behavioural activation | (Indirect evidence) Claimed effectiveness of background science | - | Yes | - |
| A18 | MoodTools - Depression Aid | CBT | (Indirect evidence) Claimed effectiveness of background science | ✓ | Yes | - |
| A19 | We are more - our support network | - | - | ✓ | N/A | - |
| A20 | Relieve Depression Hypnosis - Mood & Anxiety Help | Hypnosis | - | - | Yes | - |
| A21 | SuperBetter | Live Gamefully® method | (Direct evidence) Provided literature-based clinical effectiveness of the app | - | Yes but hard to find | - |
| A22 | T2 Mood Tracker | - | - | ✓ | N/A | Users can generate report to show clinicians |
| A23 | TalkLife | - | - | - | Yes but hard to find | - |
| A24 | The Szondi Test: Research of Depression | - | - | - | N/A | Users can generate report to show clinicians |
| A25 | ThinkUp: Positive Affirmations | Positive affirmations & self-talk | (Indirect evidence) Claimed effectiveness of background science | ✓ | Yes but hard to find | - |
| A26 | What's Up? - Mental Health App | CBT, ACT | - | ✓ | N/A | - |
| A27 | Wysa: stress, depression & anxiety therapy chatbot | CBT, DBT | (Direct evidence) Provided literature-based clinical effectiveness of the app | ✓ | Yes but hard to find | Users can activate standby coaches or therapists in app. |
| A28 | Youper - Anxiety & Depression | CBT, ACT, mindfulness | (Indirect evidence) Claimed effectiveness of background science | ✓ | Yes but hard to find | - |
| A29 | 🇬🇧Depression Test | - | - | ✓ | Yes | - |
