# Supplementary material for: Functionality of Top-Rated Mobile Apps for Depression: Systematic Search and Evaluation
Source: JMIR Ment Health. 2020 Jan 24;7(1):e15321. doi: 10.2196/15321 (PMC7007593; doi:10.2196/15321)
Supplement: Multimedia Appendix 4 [file mental_v7i1e15321_app4.docx]

| App_ID | App_name | Safety design |
| --- | --- | --- |
|  |  |  |
| A1 | Aware: Meditation & Mindfulness | - |
| A2 | Breathe Easy | - |
| A3 | CBT Thought Record Diary | - |
| A4 | Cognitive Diary CBT Self-Help | Provide link to suicide helpline |
| A5 | Depression CBT Self-Help Guide | Provide link to suicide helpline |
| A6 | Depressive and sad wallpaper | - |
| A7 | Disappointment Quotes | - |
| A8 | eMoods Bipolar Mood Tracker | Suggest to call medical emergency number |
| A9 | Fight Depression Naturally | - |
| A10 | Hypnosis for Anxiety, Stress Relief & Depression | - |
| A11 | InnerHour - Self Help for Anxiety & Depression | - |
| A12 | Lonely Wallpaper | - |
| A13 | MindCare: mental well-being analytics made easy | - |
| A14 | Mood Log | - |
| A15 | MoodKit - Mood Improvement Tools | - |
| A16 | Moodpath - Depression & Anxiety Test | Provide direct link to counselling website and suicide helpline |
| A17 | MoodSpace | - |
| A18 | MoodTools - Depression Aid | Construct personalized safety plan |
| A19 | We are more - our support network | - |
| A20 | Relieve Depression Hypnosis - Mood & Anxiety Help | - |
| A21 | SuperBetter | - |
| A22 | T2 Mood Tracker | - |
| A23 | TalkLife | Provide link to access support service |
| A24 | The Szondi Test: Research of Depression | - |
| A25 | ThinkUp: Positive Affirmations | - |
| A26 | What's Up? - Mental Health App | - |
| A27 | Wysa: stress, depression & anxiety therapy chatbot | Provide link to suicide helpline |
| A28 | Youper - Anxiety & Depression | - |
| A29 | 🇬🇧Depression Test | Suggest to call medical emergency number |
