# Supplementary material for: Functionality of Top-Rated Mobile Apps for Depression: Systematic Search and Evaluation
Source: JMIR Ment Health. 2020 Jan 24;7(1):e15321. doi: 10.2196/15321 (PMC7007593; doi:10.2196/15321)
Supplement: Multimedia Appendix 5 [file mental_v7i1e15321_app5.docx]

| Purpose of screening | App_ID | App_name | Screening material (whether validated) | Screening frequency | Feedback upon informing screening result | Provides link to suicide helpline |
| --- | --- | --- | --- | --- | --- | --- |
|  |  |  |  |  |  |  |
| Monitoring symptoms | A28 | Youper - Anxiety & Depression | PHQ-9 (validated) | Periodic | Predefined psychoeducational article | - |
|  | A18 | MoodTools - Depression Aid | PHQ-9 (validated) | Momentary but with suggestion of periodic usage | Provide self-help tips based on users' result (i.e., suicidal thoughts management, or sleeping tips) | ✔ |
|  | A27 | Wysa: stress, depression & anxiety therapy chatbot | PHQ-9 (validated) | Periodic | Predefined psychoeducational article, guides for mindfulness exercise | ✔ |
|  | A5 | Depression CBT Self-Help Guide | PHQ-9 (validated) | Momentary | Predefined psychoeducational article | ✔ |
| Self-diagnosing | A29 | 🇬🇧Depression Test | Not claimed (unknown) | Momentary | Suggests that users contact a clinician | - |
|  | A24 | The Szondi Test: Research of Depression | Szondi Test (not validated) | Momentary | - | - |
|  | A16 | Moodpath - Depression & Anxiety Test | ICD-10 (validated) | Periodic | Psychoeducation articles | - |
| Basis for personalization | A11 | InnerHour - Self Help for Anxiety & Depression | Not claimed (unknown) | Momentary | 28 days personalized intervention. | - |
|  | A19 | We are more - our support network | Users’ self-report (unknown) | Momentary | Personalize app content based on users’ disorder and symptoms. | - |
