# Supplementary material for: Functionality of Top-Rated Mobile Apps for Depression: Systematic Search and Evaluation
Source: JMIR Ment Health. 2020 Jan 24;7(1):e15321. doi: 10.2196/15321 (PMC7007593; doi:10.2196/15321)
Supplement: Multimedia Appendix 6 [file mental_v7i1e15321_app6.docx]

| App_ID | App_name | Behaviors | Thoughts | Moods | Screening results |
| --- | --- | --- | --- | --- | --- |
|  |  |  |  |  |  |
| A1 | Aware: Meditation & Mindfulness | For visualizing intervention progress |  |  |  |
| A3 | CBT Thought Record Diary |  | For thought tracking | For thought tracking |  |
| A4 | Cognitive Diary CBT Self-Help |  | For thought tracking | For thought tracking |  |
| A5 | Depression CBT Self-Help Guide |  | For thought tracking | For thought tracking | For symptom monitoring |
| A8 | eMoods Bipolar Mood Tracker |  | For thought tracking; with no template | For understanding mood patterns; track alongside physical conditions |  |
| A11 | InnerHour - Self Help for Anxiety & Depression | For visualizing intervention progress |  | For understanding mood patterns; also track mood triggering factors |  |
| A13 | MindCare: mental well-being analytics made easy | For visualizing intervention progress | For thought tracking; with no template | For understanding mood patterns; track alongside mood triggering factors and physical conditions |  |
| A14 | Mood Log |  |  | For understanding mood patterns; track alongside physical conditions |  |
| A15 | MoodKit - Mood Improvement Tools | For visualizing intervention progress | For thought tracking | For thought tracking |  |
| A16 | Moodpath - Depression & Anxiety Test |  | For thought tracking | For thought tracking | For symptom monitoring |
| A17 | MoodSpace | For visualizing intervention progress | For thought tracking | For thought tracking |  |
| A18 | MoodTools - Depression Aid | For visualizing intervention progress | For thought tracking | For thought tracking | For symptom monitoring |
| A21 | SuperBetter | For visualizing intervention progress |  |  |  |
| A22 | T2 Mood Tracker |  | For thought tracking; with no template | For understanding mood patterns; track alongside physical conditions |  |
| A23 | TalkLife |  | For thought tracking | For thought tracking |  |
| A24 | The Szondi Test: Research of Depression |  |  |  | For symptom monitoring |
| A26 | What's Up? - Mental Health App |  | For thought tracking | For thought tracking |  |
| A27 | Wysa: stress, depression & anxiety therapy chatbot | For visualizing intervention progress | For thought tracking | For thought tracking, and understanding mood patterns; also track mood triggering factors |  |
| A28 | Youper - Anxiety & Depression |  | For thought tracking | For thought tracking, and understanding mood patterns; also track mood triggering factors | For symptom monitoring |
