# Supplementary material for: Functionality of Top-Rated Mobile Apps for Depression: Systematic Search and Evaluation
Source: JMIR Ment Health. 2020 Jan 24;7(1):e15321. doi: 10.2196/15321 (PMC7007593; doi:10.2196/15321)
Supplement: Multimedia Appendix 7 [file mental_v7i1e15321_app7.docx]

| App_ID | App_name | Thought diaries | Psychoeducation | Mindfulness | Scheduling positive behaviors | Emotional expression | Other |
| --- | --- | --- | --- | --- | --- | --- | --- |
|  |  |  |  |  |  |  |  |
| A1 | Aware |  |  | Yes (meditation) |  |  |  |
| A2 | Breathe Easy |  |  | Yes (breathing guidance) |  |  |  |
| A3 | CBT Thought Record Diary | Yes (adaptive template) |  |  |  |  |  |
| A4 | Cognitive Diary CBT Self-Help | Yes (adaptive template) | Yes (generic) |  |  |  |  |
| A5 | Depression CBT Self-Help Guide | Yes (adaptive template) | Yes (personalized) | Yes (meditation) |  |  |  |
| A6 | Depressive and sad wallpaper |  |  |  |  | Yes (consuming art-based materials) |  |
| A7 | Disappointment Quotes |  |  |  |  | Yes (consuming art-based materials) |  |
| A9 | Fight Depression Naturally |  | Yes (generic) | Yes (meditation) |  |  |  |
| A10 | Hypnosis for Anxiety, Stress Relief & Depression |  |  |  |  |  | Yes (hypnosis) |
| A11 | InnerHour - Self Help for Anxiety & Depression |  | Yes (generic) | Yes (meditation) | Yes |  |  |
| A12 | Lonely Wallpaper |  |  |  |  | Yes (consuming art-based materials) |  |
| A13 | MindCare: mental well-being analytics made easy |  |  |  |  |  |  |
| A15 | MoodKit - Mood Improvement Tools | Yes (adaptive template) |  |  | Yes |  |  |
| A16 | Moodpath - Depression & Anxiety Test |  | Yes (personalized) | Yes (meditation) |  |  |  |
| A17 | moodspace | Yes (generic template) |  | Yes (meditation) |  |  |  |
| A18 | MoodTools - Depression Aid | Yes (adaptive template) | Yes (generic) | Yes (meditation) | Yes |  |  |
| A19 | We are more - our support network |  |  |  |  | Yes (online support groups) |  |
| A20 | Relieve Depression Hypnosis - Mood & Anxiety Help |  |  |  |  |  | Yes (hypnosis) |
| A21 | SuperBetter |  |  |  | Yes |  |  |
| A23 | TalkLife |  |  |  |  | Yes (online support groups) |  |
| A25 | ThinkUp: Positive Affirmations |  |  |  |  |  | Yes (positive affirmation) |
| A26 | What's Up? - Mental Health App | Yes (generic template) | Yes (generic) | Yes (grounding techniques) |  |  |  |
| A27 | Wysa: stress, depression & anxiety therapy chatbot | Yes (adaptive template) | Yes (personalized) | Yes (meditation) |  |  |  |
| A28 | Youper - Anxiety & Depression | Yes (adaptive template) | Yes (personalized) | Yes (meditation) |  |  |  |
